# Human immunodeficiency virus type-1 episomal cDNA in semen

**DOI:** 10.1186/1742-6405-2-9

**Published:** 2005-10-11

**Authors:** Chong Xu, Joseph A Politch, Kenneth H Mayer, Deborah J Anderson

**Affiliations:** 1 Division of Reproductive Biology, Department of Obstetrics and Gynecology, Boston University School of Medicine, Boston, MA 02118, USA; 2 Fenway Community Health Center, Boston, MA 02115, USA; 3 Department of Medicine, Brown University Medical School, Providence, RI 02912, USA

## Abstract

**Background:**

Episomal 2-long terminal repeat (LTR) HIV-1 cDNA, a by-product of HIV-1 infection, is used in clinical trials as a marker for ongoing viral replication. It would be useful to employ 2-LTR cDNA to monitor cryptic HIV-1 infection in the genital tract of men on antiretroviral therapy (ART) to predict the evolution of sexually transmissible drug-resistant HIV-1, but studies thus far have failed to detect this marker in semen. The objectives of this study were: 1) to use a technique that maximizes DNA recovery from HIV-1 infected white blood cells in semen to determine if episomal 2-LTR cDNA is detectable in semen of ART-naïve men with other evidence of genital tract HIV-1 infection, and 2) to compare levels of HIV-1 2-LTR cDNA, RNA, and proviral DNA in semen from HIV-1+ men on ART.

**Results:**

Using a somatic cell DNA extraction technique, 2-LTR cDNA was detected by PCR/ELISA in 4 out of 8 semen samples from ART-naïve men selected for other signs of seminal HIV-1 infection (positive controls). Southern blot and DNA sequencing confirmed that the amplified sequences were HIV-1 2-LTR cDNA; copy numbers ranged from 55 to 504 copies/sample. Two semen samples from a cohort of 22 HIV-1-infected men on dual nucleoside therapy, one with and one without detectable seminal HIV-1 RNA, were 2-LTR cDNA positive (336 and 8,560 copies/sample). Following addition of indinavir to the therapy regimen, no semen samples from 21 men with controlled peripheral and seminal viral loads were 2-LTR cDNA positive at 1 and 6 month time points, despite the persistence of HIV-1 proviral DNA+ semen cells and seminal cytomegalovirus (CMV) shedding in some cases. However, one individual who failed indinavir therapy and later developed distinct protease inhibitor (PI) drug resistance mutations in semen, maintained elevated levels of HIV-1 RNA and 2-LTR cDNA in semen.

**Conclusion:**

2-LTR HIV-1 cDNA is detectable in semen of HIV-1-infected men. Two men on ART had 2-LTR HIV-1 cDNA in semen, suggesting that this marker may prove to be useful to monitor HIV-1 infection in the genital tract of men on ART to predict the evolution of drug resistance mutations in semen.

## Background

The global AIDS epidemic is primarily attributed to the sexual transmission of Human Immunodeficiency Virus type-1 (HIV-1), a retrovirus that infects CD4+ T cells and macrophages and causes severe immunosuppression in most untreated infected individuals. In HIV-1-infected men, sexual transmission rates are high during acute infection and late-stage disease when HIV-1 viral loads are elevated in both semen and peripheral blood [1,2]. However, a number of studies have also described elevated HIV-1 levels in semen from men with low or undetectable HIV-1 concentrations in peripheral blood [3-5], and others have documented genotypic differences between seminal and peripheral blood HIV-1 [6-8]. These data indicate that the male genital tract is a compartment, like the central nervous system, in which HIV-1 replication and divergent evolution can occur under the influence of local factors. The male genital tract has several distinctive features that can affect local HIV-1 infection processes: 1) the distal portion of the male genital tract, especially the penile urethra, is capable of mounting a mucosal immune response including production of secretory IgA [9] and epithelial cell-derived mediators of innate immunity which can suppress HIV-1 infection [10]; 2) co-infection with other sexually transmitted pathogens can create inflammatory conditions that lead to increased levels of infectious HIV-1 in semen [11]; 3) the blood-testis barrier and locally produced immunosuppressive factors may protect HIV-1 from local immune responses in certain regions of the male genital tract [12], and 4) some antiretroviral drugs may not adequately penetrate regions of the male genital tract [13]. It is essential to better understand HIV-1 infection and persistence in the male genital tract in order to develop better intervention strategies to prevent the sexual transmission of HIV-1.

A circular episomal HIV-1 DNA fragment, 2-long terminal repeat (LTR) complementary DNA (cDNA), has been used as a marker for HIV-1 infection in vivo. 2-LTR cDNA is a by-product of HIV-1 infection, formed after reverse transcription of HIV-1 RNA and prior to integration of the HIV-1 cDNA genome into host cell DNA [14]. Nonintegrated HIV-1 DNA has gained clinical importance because its accumulation within infected cells is thought to indicate recent HIV-1 replication [14-16], and because its presence has been associated with cytopathic effects [17,18]. Although the stability and half-life of 2-LTR extrachromosomal HIV-1 DNA is a matter of some debate [19,20], a recent clinical study, showing that patients on suboptimal therapy acquire drug resistance mutations in episomal viral cDNA while provirus remains unchanged, reaffirms the clinical importance of this marker [21]. 2-LTR cDNA has been detected in blood and tissues of individuals on highly active antiretroviral therapy (HAART), and may be an indication of cryptic HIV-1 replication [16]. This marker is used to monitor residual viral replication and potential evolution of drug resistance mutations in viral reservoir sites in individuals on HAART [22].

Whereas effective antiretroviral therapy (ART) suppresses HIV-1 viral load in both blood and semen, HIV-1 proviral DNA often persists in semen cells [23,24]. 2-LTR cDNA could provide a useful marker for HIV-1 infection in the male genital tract, including cryptic HIV-1 replication in the genital tract in men on HAART. A recent study measured 2-LTR cDNA in blood and semen of men on HAART, but failed to detect any positive semen samples [25], possibly due to a cell selection step that can reduce the sensitivity of HIV-1 DNA detection in semen. We have applied an extraction approach that does not require cell separation and results in selective recovery of DNA from semen somatic cells including all HIV-1 infected white blood cells (WBC). The first goal of our study was to quantify 2-LTR cDNA in semen from ART-naïve men with other signs of HIV-1 infection in the genital tract (positive controls). Our second goal was to assess levels of 2-LTR cDNA in semen from men on ART.

## Materials and methods

### Semen processing

The semen samples used in this study were archived from previously published studies [1,23]. Positive control samples used for validation of 2-LTR cDNA detection/quantification were from ART-naïve men with evidence of seminal HIV-1 viremia (Group I). HIV-1 proviral DNA in the cellular fraction had been assessed by quantitative PCR; infectious HIV-1 was detected in seminal plasma and cellular fractions by p24 assay following up to 21 days of coculture with phytohaemagglutinin A (PHA)-stimulated peripheral blood mononuclear cells (PBMC) [1]. These samples were collected before RT-PCR was widely available, so HIV-1 RNA copy numbers were not assessed. Samples used to test for seminal 2-LTR cDNA (cryptic HIV-1 replication) in men on antiretroviral therapy were from 22 HIV-1-positive men before and at 1 and 6 months after addition of indinavir to dual nucleoside analog therapy (Group II). Clinical information for this cohort has been presented in an earlier report (23). Seminal white blood cell counts were obtained at the time of semen collection by microscopic analysis of peroxidase-stained polymorphonuclear neutrophils (PMN) and differential counts of lymphocytes and macrophages by immunohistology [26]. Semen from this group was tested for HIV-1 proviral DNA and RNA by quantitative polymerase chain reaction (PCR)/reverse transcriptase (RT)-PCR and blood viral loads were monitored by the Chiron branched DNA RNA-1 assay [23]. For 2-LTR cDNA assessment, aliquots containing cell pellets from 250 μL of semen that had been stored at -80°C were thawed. Nonspermatozoal semen cells were lysed in buffer containing 1% SDS and 100 μg/mL proteinase K. DNA was extracted with the standard phenol/chloroform/ethanol procedure, and stored in distilled water at 4°C until use.

### 2-LTR cDNA detection and quantification

The 2-LTR cDNA sequence was amplified by PCR in the presence of 10 nM digoxigenin (DIG)-labeled dUTP (Roche Diagnostics, Indianapolis, IN), to label the PCR-amplified DNA products with DIG for their subsequent ELISA analysis. The PCR cycling conditions were: 95°C for 10 min, 40 cycles at 95°C for 30 sec, 57°C for 30 sec and 72°C for 30 sec. The sense and antisense primers were: 5'-AACTA GGGAA CCCAC TGCTT AAG-3' and 5'-TCCAC AGATC AAGGA TATCT TGTC-3'[27]. Positive controls (PBMC from HIV-1-infected men) and negative controls (PBMC and semen from uninfected men) were included in each experiment and underwent the same processing as the test samples.

For the ELISA and Southern blot assays, a 29-base biotinylated probe was constructed (5'-GGAAA ATCTC TAGCA GTACT GGAAG GGCT-3') containing, successively, the last 15 bases of the HIV-1 genome (bases 9705–9719), an additional 4 bases, GTAC, specifically found at HIV-1 2-LTR circle junction, and the first 10 bases of the HIV-1 genome (bases 1–10). In ELISA, DIG-labeled PCR products were bound to the streptavidin-coated microtiterplate (Roche Diagnostics, Indianapolis, IN) via the biotinated probe during a 3 hour-incubation at 56°C, and reacted with anti-DIG antibody-HRP conjugate. Optical density (OD) was measured at 405 nm after adding the HRP substrate ABTS. Samples were considered positive if the OD_405 _was higher than the average of the negative PCR controls plus 3 times standard deviation (OD_neg _+ 3 SD). Copy numbers of 2-LTR cDNA were determined from a standard curve obtained by amplification of serially-diluted 2-LTR cDNA positive control amplicons. As few as 10 copies of 2-LTR c DNA could be consistently detected in repeated runs. For each sample, a housekeeping gene, glyceraldehyde-3-phosphate dehydrogenase (GAPDH), was also amplified and quantified by ELISA to verify the presence of amplifiable DNA.

The PCR products were also analyzed by agarose gel electrophoresis, Southern blot and DNA sequencing. Fifteen microliters of PCR product were separated on a 1% agarose gel and transferred to a Hybond N+ membrane (Schleicher & Schuell, Keene, NH); Southern blot was performed using the 29-base biotinylated probe. After a high-stringency wash, streptavidin-HRP conjugate and its substrate in Luminol/enhancer solution (Pierce, Rockford, IL) were added to the membrane. The image was acquired with Fluorchem SP (Alpha Innotech, San Leandro, CA). Direct sequencing of the PCR product was performed by the Dana-Farber/Harvard Cancer Center core facility.

### Cytomegalovirus (CMV) detection

Because CMV infection is common in HIV-1 infected men, and seminal shedding of CMV has been associated with elevated levels of HIV-1 in semen [28], we assayed semen cell extracts for CMV DNA by PCR/ELISA. The sense and antisense primers for CMV were: AGGCG TGTAC GGTGG GAGGT CT and CCGCG TTCCA ATGCA CCGTT CC [29]. The biotin-labeled probe was CCATA GAAGA CACCG GGACC GATCC AGCCT. The PCR parameters were 95°C 30 sec and 60°C 1 min for 40 cycles.

## Results

2-LTR cDNA was detected in semen from 4/8 men in Group I (positive controls). Copy numbers ranged from 55 to 504 copies/sample (Table 1). Semen from 2/22 men in Group II on dual nucleoside analog ART were positive for 2-LTR cDNA (336 and 8,560 copies/sample); one of the 2-LTR cDNA positive samples had undetectable HIV-1 RNA, the other had elevated HIV RNA (4 × 10^5 ^copies, Table 1). Following the addition of the protease inhibitor (PI), indinavir, to the ART regimen, 0/40 semen samples from 21 men with peripheral and seminal HIV-1 RNA suppression were positive for 2-LTR cDNA (all 21 tested at one-month, and 19 retested at 6 months). The remaining subject in Group II, patient A, was intolerant of the indinavir regimen (nausea) and missed several doses. By one month, blood HIV-1 RNA remained undetectable for this individual, but seminal HIV-1 RNA copy numbers increased from 4 × 10^5 ^(baseline) to 2 × 10^6^. He was positive for seminal 2-LTR cDNA while on dual nucleoside therapy before indinavir therapy (8,560 copies/sample), and levels increased at the 1-month post indinavir treatment time point to 20,944/sample (Table 1). At 5-months after initiation of indinavir, his treatment regimen was changed (stavudine, sequinavir and continued lamivudine); by the 6-month time point blood viremia remained undetectable, semen HIV-1 RNA decreased to 3 × 10^5^, and 2-LTR cDNA was undetectable. Of interest, we previously reported that distinct PI resistance mutations were detectable in semen and blood from Patient A 18 months after the failed indinavir therapy (23).

**Table 1:** 2-LTR cDNA Positive Semen Samples

**Group**	**ID#**	**Antiretroviral Therapy**	**Seminal HIV-1** **2-LTR cDNA^a^**	**Seminal proviral** ** HIV-1 DNA**	**Seminal HIV-1 RNA^a ^** **or Infectious HIV-1**	**Seminal CMV DNA**	**Seminal HIV** **Host Cells^b^**
I	1	-	504	+	- (Inf)	+	ND
I	2	-	55	+	+ (Inf)	+	1.1 × 10^6^
I	3	-	430	+	+ (Inf)	-	ND
I	4	-	70	+	? (Inf)	-	ND
II	A-0^c^	ZVD, 3TC	8,560	+	400,000	+	5.1 × 10^6^
II	A-1^d^	ZVD, 3TC, IDV	20,944	+	2,000,000	+	3.6 × 10^5^
II	B-0	ZVD, 3TC	336	+	0	+	2.6 × 10^6^

^a ^Expressed as copy number per sample

^b ^Sum of macrophages and CD4+ lymphocytes/sample

^c ^Baseline

^d ^After 1 month on protease inhibitor therapy

**Figure 1:**
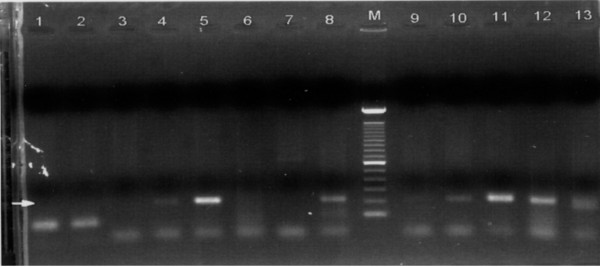
Agarose gel electrophoresis of HIV-1 2-LTR cDNA amplified from human semen. The arrow indicates the 2-LTR cDNA bands. Lanes 5 and 11 are positive controls. Lane M is a DNA ladder. Lanes 4, 8, 10, 12, 13 are positive semen samples.

**Figure 2:**
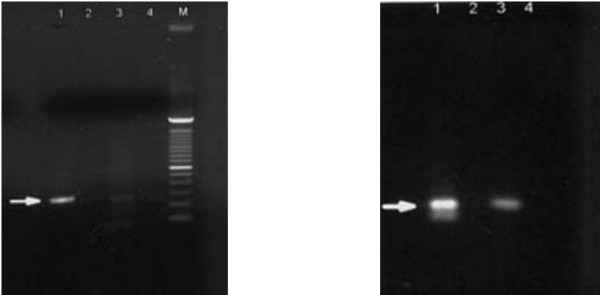
Southern blot (right panel) confirmation of seminal HIV-1 2-LTR cDNA transferred from agarose gel (left panel, lane 3)

2-LTR cDNA from the four positive samples with the strongest signals in PCR/ELISA were also detected as 190 bp bands on agarose gels, confirming the correct size of the amplified products (Figure 1). The binding of the biotinylated probe to the PCR product in Southern blot assay verified the correct orientation of the PCR amplicon. (Figure 2). To confirm that HIV-1 2-LTR cDNA had been amplified, PCR products from four 2-LTR cDNA positive semen samples and positive PBMC controls were purified and directly sequenced. The sequence results showed that the PCR products were the expected fragment containing the exact sequence spanning the HIV-1 genome base numbers 9585 to 9719 and 1 to 51.

All seven of the 2-LTR cDNA positive semen samples showed other indications of HIV-1 infection. Four of the 2-LTR cDNA+ semen samples from Group I were from PI-naïve men with leukocytospermia (LCS, i.e., ≥1,000,000 WBC/mL semen) and high seminal HIV-1 proviral DNA copy numbers. Two out of three of the Group I 2-LTR cDNA+ samples also contained cell-free infectious HIV-1; the fourth 2-LTR cDNA+ was indeterminate in the culture assay due to bacterial contamination. Seminal HIV-1 RNA levels were not available for Group I. All three of the 2-LTR cDNA positive semen samples from Group II men contained proviral DNA; one was leukocytospermic, and the two samples from Patient A at baseline and one-month also contained high concentrations of HIV-1 RNA. Five out of seven of the 2-LTR cDNA+ semen samples (Groups I and II) were also positive for CMV DNA (Table 1).

On the other hand, many semen samples with indicators of HIV-1 infection were negative in the 2-LTR cDNA assay. Of the four negative Group I samples, all were from ART-naïve men with seminal HIV-1 proviral DNA (inclusion criteria for Group I); one was culture-positive for HIV-1, and 2 were CMV DNA+. Of the 20 2-LTR cDNA-negative Group II baseline samples, one was leukocytospermic, 4 had >100 copies of HIV-1 proviral DNA, 5 contained >1,000 copies of cell-free HIV-1 RNA and 6 were CMV DNA-positive. Of the 40 2-LTR cDNA-negative post-indinavir semen samples, four were leukocytospermic, 12 had proviral HIV-1 DNA, and 5 were from men with seminal CMV DNA.

## Discussion

This study demonstrates for the first time that HIV-1 2-LTR cDNA, a clinical marker of HIV-1 infection, can be detected in semen from HIV-1-infected men. Because seminal HIV-1 RNA can potentially originate from outside the genital tract, and seminal HIV-1 proviral DNA may represent latent infection, 2-LTR cDNA may prove to be a valuable marker for monitoring HIV-1 infection within genital tract tissues. The highest copy numbers of 2-LTR cDNA in this study were detected in semen of a man failing indinavir therapy who later developed unique PI resistance mutations in semen. Data from this study suggest that seminal 2-LTR cDNA may provide a useful marker to predict the evolution of sexually transmissible HIV-1 drug resistance mutations in men on ART.

Since the number of HIV-1-infected cells in semen is restricted by small sample size, steps must be taken to maximize HIV-1 DNA extraction. In the only other published study to investigate 2-LTR cDNA in semen, Nunnari et al. [25] isolated seminal WBC on Ficoll before DNA extraction and 2-LTR c-DNA assay. This procedure eliminates HIV-1-infected macrophages, which are an important source of infectious virus, and also reduces the T cell yield [30]. Therefore, the sensitivity of 2-LTR cDNA detection in their study may have been compromised. In our study, we used a direct lysis technique to extract DNA from nonspermatozoal cells in semen cell pellets. Sperm DNA is tightly bound by disulphide bonds in histones, and its extraction requires the addition of a reducing agent such as dithiothreitol (DTT) [31]. Our lysis technique leaves the sperm heads intact and provides extracts enriched for WBC DNA. In this study, numbers of semen WBC ranged from 1.8 × 10^5 ^to 2.7 × 10^6^/mL for 2-LTR cDNA-positive samples, and 2-LTR cDNA copy numbers were relatively low (range 10–630 per 10^6 ^cells). The sensitivity of 2-LTR cDNA detection in semen could be further increased by using a larger fraction of the semen sample, instead of a small aliquot as was used in this study, and by centrifugation procedures that enrich episomal HIV-1 DNA prior to the PCR reaction. Cell fractionation procedures could be applied to samples with high copy numbers of 2-LTR cDNA to identify cellular targets of HIV-1 infection in the male genital tract.

All of the 2-LTR cDNA+ semen samples contained HIV-1 proviral DNA, and all but two were positive for HIV-1 RNA or infectious virions. Five out of 7 of the 2-LTR cDNA+ semen samples were also positive for seminal CMV DNA, but this association was not statistically significant because CMV infection was common overall (4/8 men in Group I and 9/22 men in Group II were positive for seminal CMV).

Two men on dual nucleoside analog treatment had 2-LTR cDNA in semen; one of these individuals failed subsequent indinavir therapy and continued to have high levels of HIV-1 RNA and 2-LTR cDNA in semen, although peripheral viral load was undetectable. These data provide further evidence that HIV-1 replication can occur in the genital tract of men on ART, potentially leading to the evolution of drug resistance mutations in semen that cannot be monitored in peripheral blood. On the other hand, none of 40 post-indinavir semen samples from men controlling HIV-1 RNA levels in blood and semen was positive for 2-LTR cDNA, suggesting that cryptic HIV-1 replication in the genital tract (without the appearance of HIV-1 RNA in blood or semen) may be uncommon in men on effective ART.

The origin of HIV-1 and infected cells in semen is poorly understood. Some infected cells and HIV-1 virions in semen may originate from infection outside the genital tract, but genetic evidence indicates that at least some of the HIV-1 detected in semen is distinct from HIV-1 in peripheral blood [6-8]. Although germ cells and other cell types have been studied as potential HIV-1 reservoirs in the male genital tract, recent attention has focused on genital tract WBC. Semen contains variable numbers of CD4+ T cells and macrophages [32], and recent studies using immunobeads or gradient/sperm-wash methods have demonstrated that these semen cell types carry HIV-1 proviral DNA and are highly infectious [30,33]. HIV-1-infected T cells and macrophages have been detected throughout the male genital tract [34]. Inflammatory conditions, such as those occurring during infection with other sexually transmitted pathogens, attract large numbers of lymphocytes and macrophages into the seminal compartment, and are associated with higher numbers of infected cells and infectious virus in semen [1,11,35]. It is unknown to what extent HIV-1 infected WBC migrate to the genital tract from peripheral blood or other tissues, or are infected in situ by HIV-1 in the genital tract. A recent study entailing phylogenetic analysis of nucleotide sequences of the C2-V5 region of HIV-1 gp120 from HIV-1 RNA isolated from semen cells and seminal plasma from 5 HIV-1-infected men provided evidence that cell-free HIV-1 in semen has distinct sequences and may not be derived from cells represented in semen [36]. Future studies on the presence of HIV 2-LTR cDNA in genital tract and semen cell populations should provide improved insight into sources of HIV-1 infection in the male genital tract.

## Conclusion

This study, which shows that HIV-1 2-LTR c-DNA is detectable in semen cells, provides evidence that HIV-1 infection occurs in the genital tract. This molecular marker of HIV infection could provide an important research tool for better understanding sites of HIV-1 infection in the male genital tract, and as a clinical tool for monitoring genital tract HIV-1 infection in men on suppressive ART.

## Competing interests

The author(s) declare that they have no competing interests.

## Authors' contributions

CX performed all molecular assays. JAP processed samples, performed semen and WBC analyses and maintained databases. KHM coordinated the procurement of semen and clinical information. DJA was responsible for the overall experimental design and implementation of the project. All authors contributed to the writing of the manuscript.
